# Sexual and Contraceptive Behaviour of Adolescents and Young Adults in Germany

**DOI:** 10.1093/eurpub/ckac130.191

**Published:** 2022-10-25

**Authors:** S Scharmanski, AH Hessling

**Affiliations:** Sexuality Education, Contraception and Family, Federal Centre for Health Education, Köln, Germany

## Abstract

**Background:**

For 40 years the Federal Centre for Health Education in Germany has been analysing the contraception behaviour of young people. The current Survey is the ninth iteration, carried out in 2019. This continuous monitoring generates insights on the sexual and reproductive health of young people in Germany. The survey provides an important basis for the development of sexuality education and family planning measures.

**Methods:**

A total of N = 6032 adolescents and young adults participated in the survey. Date collection was conducted by computer-assisted personal interviewing (CAPI). The current sexual and contraceptive behaviour of adolescents and young adults will be summarized using descriptive results. In addition, the association between contraception non-use and sociodemographic factors, characteristics of sexuality education and situated factors of first sexual intercourse is analysed by multivariate logistic regressions.

**Results:**

A key finding of this iteration is that with regards to the age of the first sexual intercourse, the proportion of adolescents younger than 17 years has been declining for several years. For contraception, adolescents most frequently used condoms, and use of the pill has decreased. 9% of the participants reported non-contraception use at first sexual intercourse. This is significantly associated (p < .01) with unexpected and only unilaterally desired sexual intercourse and the absence of sexuality education in School. In addition, the younger the adolescence were at first sexual intercourse the greater the risk for contraception non-use.

**Conclusions:**

The data from the current iteration indicate safe and responsible contraceptive behaviour among young people in Germany. It is important to maintain the commitment in the field of sexual health promotion and expand prevention measures for young people. This is the only way to ensure the sexual and reproductive health also in the next generation.

**Key messages:**

- Data from Youth Sexuality Study indicate safe and responsible contraceptive behaviour among young people in Germany.
- Commitment in the field of sexual health promotion needs to be continued.

